# Distribution and Diversity of Pathogenic *Leptospira* Species in Peri-domestic Surface Waters from South Central Chile

**DOI:** 10.1371/journal.pntd.0004895

**Published:** 2016-08-16

**Authors:** Meghan R. Mason, Carolina Encina, Srinand Sreevatsan, Claudia Muñoz-Zanzi

**Affiliations:** 1 Division of Epidemiology and Community Health, School of Public Health, University of Minnesota, Minneapolis, Minnesota, United States of America; 2 Institute of Preventive Veterinary Medicine, Universidad Austral de Chile, Valdivia, Chile; 3 College of Veterinary Medicine, University of Minnesota, Saint Paul, Minnesota, United States of America; Institut Pasteur, FRANCE

## Abstract

**Background:**

Leptospirosis is a neglected zoonosis affecting animals and humans caused by infection with *Leptospira*. The bacteria can survive outside of hosts for long periods of time in soil and water. While identification of *Leptospira* species from human cases and animal reservoirs are increasingly reported, little is known about the diversity of pathogenic *Leptospira* species in the environment and how surveillance of the environment might be used for monitoring and controlling disease.

**Methods and Findings:**

Water samples (n = 104) were collected from the peri-domestic environment of 422 households from farms, rural villages, and urban slums participating in a broader study on the eco-epidemiology of leptospirosis in the Los Rios Region, Chile, between October 2010 and April 2012. The *secY* region of samples, previously detected as pathogenic *Leptospira* by PCR, was amplified and sequenced. Sequences were aligned using ClustalW in MEGA, and a minimum spanning tree was created in PHYLOViZ using the goeBURST algorithm to assess sequence similarity. Sequences from four clinical isolates, 17 rodents, and 20 reference strains were also included in the analysis. Overall, water samples contained *L*. *interrogans*, *L*. *kirschneri*, and *L*. *weilii*, with descending frequency. All species were found in each community type. The distribution of the species differed by the season in which the water samples were obtained. There was no evidence that community-level prevalence of *Leptospira* in dogs, rodents, or livestock influenced pathogen diversity in the water samples.

**Conclusions:**

This study reports the presence of pathogenic *Leptospira* in the peri-domestic environment of households in three community types and the differences in *Leptospira* diversity at the community level. Systematic environmental surveillance of *Leptospira* can be used for detecting changes in pathogen diversity and to identify and monitor contaminated areas where an increased risk of human infection exists.

## Author Summary

Leptospirosis is a zoonotic disease that is caused by either direct contact with the urine of animals infected with pathogenic forms of *Leptospira*, or indirectly, through contact with contaminated water or soil. Because many people become infected through the environment, where the bacteria can live for many months if the conditions are suitable, we tested water samples from the peri-domestic area in twelve different communities from Los Rios region, Chile, to examine whether the *Leptospira* species were different in urban, rural village, and farm areas. We found that all three community types had *L*. *interrogans*, *L*. *kirschneri*, and *L*. *weilii*. No evident associations were seen between animal infection and the frequency or diversity of *Leptospira*. The proposed surveillance method has potential for systematic monitoring of surface waters that will help us better understand the importance of animal, climate, and environmental factors in the variation of *Leptospira* species present in a community in order to predict infection risk and inform prevention programs.

## Introduction

Each year, an estimated 1.03 million cases of human leptospirosis occur worldwide, resulting in 2.9 million disability adjusted life years lost [1,2]. Transmission of *Leptospira* to animal hosts and humans occurs either through direct contact with the urine of an infected animal, or indirectly, through contact between mucosae or open skin and contaminated soil or water [3,4]. The resulting clinical disease in humans can range from asymptomatic infection to flu-like symptoms, jaundice, and pulmonary hemorrhaging, with a case fatality of 10–50% [5]. Risk factors associated with human infection include specific occupational hazards and recreational activities [4,6,7], but also commonplace exposures such as living in urban areas [8], contact with livestock and companion animals in the peri-domestic environment [9], and walking barefoot [10]. Many of these factors are related to living in resource-poor settings, and indeed, the impact of leptospirosis on a community depends heavily on where it rests on the socio-economic gradient [11,12].

Many studies have documented the impact that rainfall and flooding have on the incidence of leptospirosis in humans and animals [13–17]. With lasting humidity and warm temperatures, *Leptospira* can persist in the environment for several months [3]. Studies have also previously examined the concentration and species of *Leptospira* present in surface waters in tropical areas where heavy flooding occurs seasonally. In urban sites in Malaysia, water samples collected from public spaces including lakes, swamps, and effluent drains, as well as soil samples near households, were examined for *Leptospira* using culture [18]. Approximately 23% of all samples tested positive, however, many of the species found were intermediate and saprophytic (*L*. *wolffii*, *L*. *biflexa*, and *L*. *meyeri)* [18]. In Peru, surface waters in both urban and rural areas were assessed for *Leptospira* using PCR methods, revealing increased species diversity in rural areas compared with urban centers. Urban samples were primarily identified as *L*. *interrogans* while rural area samples were primarily *L*. *santarosai* and *L*. *noguchii* [19]. Both studies recommended regular monitoring of the environment for identifying contaminated areas where humans may be at higher risk for infection.

Recent developments in PCR methods have made it possible to more efficiently determine the presence of pathogenic *Leptospira* from environmental samples [20,21]. Presently, public health responses to *Leptospira*-contaminated bodies of water are reactionary. There can be a delay of several days or weeks from the start of an outbreak to messages reaching the community advising them to avoid the contaminated sources, by which point human infection has already occurred [22]. In high risk areas, it would be beneficial to have systematic surveillance of *Leptospira* in the environment so that meaningful changes in the amount or the geographic distribution of the pathogen can be detected in advance of human cases. Additional improvements to molecular sequencing are also allowing for rapid genetic classification of *Leptospira* which can be useful for integration of animal and human surveillance [23]. Shifts in the relative abundance of particular species in these settings may be indicative of changes in the local ecology and heightened risk for human infection.

The objective of this study was to describe the presence and species diversity of *Leptospira* in surface water samples from the peri-domestic environment in a region with endemic levels of leptospirosis in humans and animals. Of particular interest was examining the differences in the molecular makeup of the pathogen across rural, small village, and urban community types.

## Materials and Methods

### Surface water sample collection

The water samples collected for this study were obtained as part of a broader research project on the eco-epidemiology of leptospirosis in the Los Rios Region of South Central Chile between November 2010 and April 2012. Valdivia, the capital of the Los Rios Region (39° 48′ 50”S, 17° 14’ 45”W), receives an average of 2500 mm of rainfall annually, and experiences temperatures between 7.6° and 16.9°C throughout the year [24]. Water samples were collected from the peri-domestic environment of 422 households in twelve communities across three community types: marginalized or urban slum communities (U, n = 142 households), rural villages (C, n = 134 households) and rural farm areas (D, n = 146 households). Households were selected randomly from within each community as part of the larger research study and were enrolled based on their willingness to participate in a household questionnaire, collection of serum samples from pets, livestock and household members, rodent trapping efforts in and around the household, and water sample collection. A detailed description of the components of this cross-sectional study has been previously provided [25,26]. Reported results from these same communities document wide evidence of *Leptospira* exposure in animals and people. Overall seroprevalence in humans was 6%, ranging by community from 3% to 10% [27]. Additionally, 26%, 16%, and 37% of dogs, sheep, and cattle were seropositive, respectively, and 20% of trapped rodents were PCR positive [26–28] (S2 Fig).

Water samples were collected primarily from the peri-domestic environment, outside of the physical housing structure and within the household’s property limits. In farm areas and villages, this included the yard, and any livestock living spaces within approximately a 15-20m radius. In urban slums, because of the close proximity between houses, the sampling space was limited to small yards separating houses. Water sample sources included pails, buckets, large bins, animal drinking troughs, trash cans, small streams, ditches, puddles, and standing water. Based on additional usage information from study participants, these sources were further classified into five categories for analysis: flowing water sources, animal drinking troughs, puddles, containers, and household drinking water sources. Study staff collected water from as many of the water sources described above within a household’s peri-domestic area, up to a limit of 7 samples per household. At least 50mL of water, 1L when possible, was collected using sterile technique and poured into a labeled Whirlpak bag [29]. Samples were stored at 4°C for no more than 24 hours before processing through previously described methods [25].

### Extraction and PCR detection of *Leptospira*

Extraction of whole DNA from water samples was conducted using a commercially available QIAamp DNA Mini Kit (Qiagen, Valencia, California, US), per the manufacturer’s instructions. DNA elution was performed with 200 μl of elution buffer. All water samples were tested using two PCR protocols, and all amplifications included a negative control, consisting of water, and a positive plasmidial control. The first PCR was a nested PCR protocol using the Lepat set of primers (Lepat1 and Lepat2) targeting the 16s rRNA gene found by Murgia et al. to identify pathogenic *Leptospira* [20]. A 510 base pair product was amplified in the first round using 16S13 (5’-CGG CGC GTC TTA AAC ATG–3’) and 16S522 (5’-TCC GCC TAC ACA CCC TTT AC-3’) primers. A second amplification round of a 330 bp product was obtained using Lepat1 (5’-GAGTCTGGGATAACTTT-3’) and Lepat2 (5’-TCACATCGYTGCTTATTTT-3’) primers. The second PCR protocol targeted a 242 base pair fragment of the *lipL32* gene that also detects pathogenic *Leptospira*. This portion of the *Leptospira* genome was amplified with the LipL32-45F (5’-AAGCATTACCGCTTGTGGTG-3’) and LipL32-286R (5’-GAACTCCCATTTCAGCGATT-3’) primers [30,31]. GoTaq Flexi DNA polymerase was used in combination (5:1) with Pfu DNA Polymerase (Promega, Madison, Wisconsin, US) for all PCR protocols.

A sample was considered positive for pathogenic *Leptospira* if it tested positive through either PCR protocol. All of these positive samples as well as DNA from 20 reference *Leptospira* strains obtained from the Royal Tropical Institute, The Netherlands (S1 Table), four local clinical isolates (two humans, one horse, and one cow), and 17 rodents (7 *Rattus rattus*, 7 *Mus musculus* and 3 wild rodents) captured from the same 422 households [26] were then tested using a PCR that targets the *secY* gene. The *secY* primers used were SecYIVF (5’- GCGATTCAGTTTAATCCTGC -3’) and SecYIV (5’- CTTAGATTTGAGCTCTAACTC -3’), with a target of 202 base pairs [32]. After amplification, the PCR products were purified and sequenced (Macrogen Inc., Seoul, Korea) and then used in a BLAST search of GenBank (National Center for Biotechnology Information, Bethesda, MD) to verify their similarity to other pathogenic *Leptospira* sequences. The chromatograms obtained from sequencing were cut at the start and end of the primers for the respective forward and reverse sequences, and examined visually for mismatches in the nucleotide bases using Sequencher 4.10 (Gene Codes Corporation, Ann Arbor, Michigan, USA). A sample was considered suitable for phylogenetic analysis if the forward and reverse sequences could be aligned, and any mismatches between base pairs could be resolved based on visual inspection of the chromatogram. Sequences are available in GenBank (Accession numbers: KX444622-KX444627, KX513514—KX513524, KX513410—KX513513).

### Statistical and phylogenetic analyses

Sample positivity was tabulated and described by community type (rural farms, rural villages, or urban slum areas), year, and season of sampling (Spring: August-November, or Summer: December-April). A mixed-effects logistic regression model with random intercepts at the community level was used to examine associations between these variables and sample PCR-positivity. Correlations between the proportion of seropositive dogs, livestock, PCR-positive rodents, and the proportion of PCR-positive water samples within a community were also calculated. Data for the community-level seroprevalence of *Leptospira* in livestock and dogs, and the PCR data for *Leptospira* carriage in rodents were obtained from the data collected for the broader study in the region [25,28] (S2 Fig).

The *secY* sequences from the PCR-positive samples were compared to the sequences from the reference strains using the goeBURST algorithm in PHYLOViZ at the single-, double- and triple-locus variant levels, corresponding to the number of base pair differences between samples [33,34]. Each water, rodent, and clinical sample was classified as a particular *Leptospira* species based on the reference sequence to which it shared the most genetic similarity. The resulting minimum spanning tree was paired with data from the water sample collection sites to visually assess associations between household characteristics and the way in which samples clustered on the minimum spanning tree. Fisher’s exact tests were used to examine whether the distribution of *Leptospira* species differed across community types (rural farms, rural villages, or urban slum areas), year, and season of sampling (Spring: August-November, or Summer: December-April) [35]. Differences in the distribution of *Leptospira* species by sample type, average temperature of the preceding seven days (°C), rainfall in the preceding 30 days (mm), and household-level prevalence of animals and rodents with *Leptospira* infection were also assessed because of their relevance in prior analyses [25,26,28].

### Ethics statement

The study protocol was approved by the University of Minnesota’s Institutional Review Board (No. 0903M62042), the Institutional Animal Care and Use Committee (No. 0904A63201), and Austral University’s Human and Animal Ethics Committee (No. 01/09). The Public Health Service Policy on Humane Care and Use of Laboratory Animals in testing, research, and training provides the core of the operational guidelines for the University of Minnesota Institutional Animal Care and Use Committee. Head of households, all adults, provided written informed consent prior to participation in this study.

## Results

### Presence of pathogenic *Leptospira*

From the 422 households in the Los Rios Region, 359 (85.1%) had at least one environmental water sample collected. In those 359 households, 816 water samples were obtained, of which, 153 (18.8%) were PCR positive for pathogenic *Leptospira* by at least one of the two PCR protocols (Table 1). Farm areas had the most water samples collected (n = 359) and also the highest proportion of PCR-positive samples (20.6%). Of the different water sample types, puddles were most commonly contaminated with pathogenic *Leptospira* (27.3%). In the mixed model with the random intercept for community, community type was not associated with whether a water sample was PCR-positive (D vs C p-value = 0.94 and U vs C p-value = 0.71). This is likely due to the distinct differences in the proportion of positive water samples at the community level, with large differences across communities of the same type. Among the rural villages, between 10.5% and 37.5% of samples tested positive, 6.1% and 43.1% of samples tested positive in rural farm areas, and between 4.9% and 33.3% in urban slum communities. The mixed regression model also indicated an interaction between season and year of sampling. In the second year of the study, more samples collected in summer (December-April) tested positive (39.3%) compared with samples collected in spring (August-November) (20.3%, p-value = 0.04). This was not observed in the samples collected during the first year (p-value = 0.77). No trends were observed between the proportion of PCR-positive water samples and the proportions of livestock or dogs seropositive for *Leptospira* or the proportion of PCR-positive rodents (S2 Fig). Seropositivity ranged from 4.5% -22.6% for sheep, 10.7%-75.0% for cows, and 4.8%-68.0% for dogs. The proportion of PCR- positive rodents ranged from 0–41% at the community level.

**Table 1:** *Leptospira* PCR results by community type and sample characteristics for water samples collected from households in the Los Rios Region, Chile (2010–2012).

Sample Characteristic	Rural Village (C) n = 217	Farm Area (D) n = 359	Urban Slum (U) n = 240	Total n = 816
	PCR+ (%)	PCR+ (%)	PCR+ (%)	PCR+ (%)
**Subtotals**	39/217 (18.0%)	74/359 (20.6%)	40/240 (16.7%)	153/816 (18.8%)
**Sample Type**				
Animal drinking trough	5/28 (17.9%)	9/55 (16.4%)	0/2 (0%)	14/85 (16.5%)
Puddle	18/84 (21.4%)	35/104 (33.7%)	31/120 (25.8%)	84/308 (27.3%)
Human drinking water	0/10 (0%)	11/47 (23.4%)	3/38 (7.9%)	14/95 (14.7%)
Flowing source	5/45 (11.1%)	6/62 (9.7%)	2/27 (7.4%)	13/134 (9.7%)
Container	11/50 (22.0%)	13/91 (14.3%)	4/53 (7.5%)	28/194 (14.4%)
**Year of Sample**				
2010–2011 (Year 1)	13/84 (15.5%)	15/176 (8.5%)	9/120 (7.5%)	37/380 (9.7%)
2011–2012 (Year 2)	26/133 (19.6%)	59/183 (32.2%)	31/120 (25.8%)	116/436 (26.6%)
**Season of Sample**				
Spring (August–November)	18/120 (15.0%)	28/111 (25.2%)	24/168 (14.3%)	70/399 (17.5%)
Summer (December–April)	21/97 (21.6%)	46/248 (18.5%)	16/72 (22.2%)	83/417 (19.9%)
**Presumptive Species**				
*L*. *interrogans*	10 (62.5%)*	46 (80.7%)*	22 (71.0%)*	78 (75.0%)*
*L*. *kirschneri*	4 (25.0%)	5 (8.8%)	7 (22.6%)	16 (15.4%)
*L*. *weilii*	0 (0%)	2 (3.5%)	1 (3.2%)	3 (2.9%)
Unclassified *Leptospira sp*. ^†^	2 (12.5%)	4 (7.0%)	1 (3.2%)	7 (6.7%)

* Distribution of species among sequences obtained in each community type.

^**†**^ Unclassified *Leptospira sp*. are sequences that were confirmed to be pathogenic *Leptospira* in a search of the NCBI database, but had more than a three base pair difference from reference strains used in the phylogenetic analysis

### *Leptospira* species from water samples

**Fig 1:**
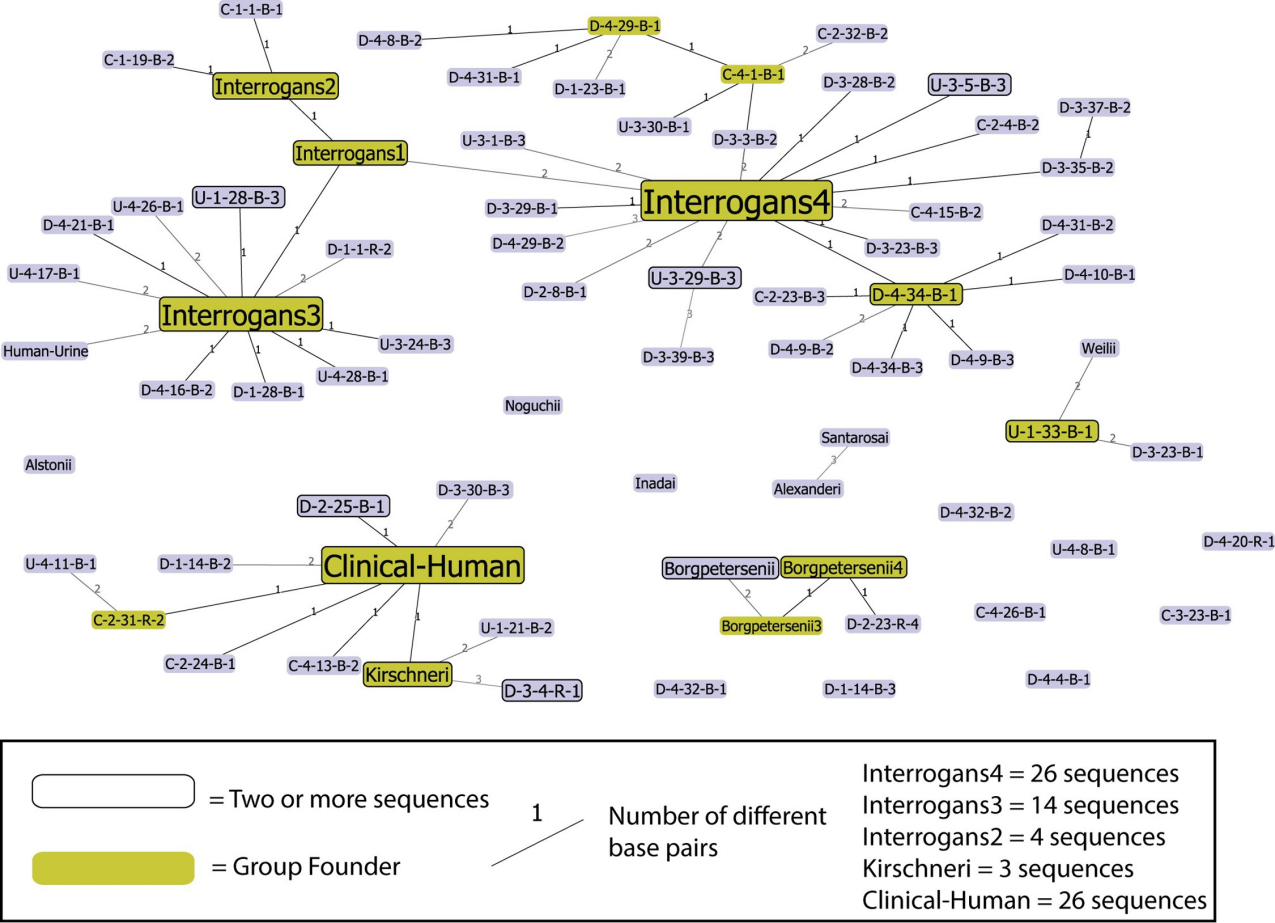
Full Minimum Spanning Tree of sequences from surface water samples (n = 104), reference strains (n = 20), rodent kidney (n = 17), and livestock and human clinical samples (n = 4), from the Los Rios Region, Chile, 2010–2012 using the *secY* (202 bp) gene. Green rectangles represent group founders and rectangles with a black border indicate nodes where multiple samples had identical sequences (two sequences each, unless otherwise noted in the legend). Samples labeled with a *Leptospira* species name are reference strains. For the water and rodent samples, the first letter represents the community type of origin (C = rural village, D = farm area, U = urban slum), the first number indicates the community number (1–4 for each community type), followed by the household number. The second letter indicates whether it is a water (B) or rodent (R) sequence and the last number corresponds to the sample number from a particular household. The clinical samples not already identical to other sequences and absorbed into the larger rectangles are listed by their types: Clinical-Human (blood sample) and Human-Urine.

From the 153 PCR positive samples, 104 (12.7% of all water samples) had a sequence suitable for phylogenetic analysis using the *secY* gene (S1 Fig). In the goeBURST analysis of the sequences from the *secY* gene, all sequences were not connected in the network until 18 variant loci were considered. Because the DNA from water samples were not obtained from isolates, some variation in the genetic makeup of the sequences relative to the reference sequences was expected, and therefore the triple-variant locus Minimum Spanning Tree (MST) was used for classification of samples. Fewer than three base pair differences from a reference sequence were considered to be indicative of the correspondent species. Based on this classification, the most common *Leptospira* species in water samples was *L*. *interrogans* (75.0%, n = 78), followed by *L*. *kirschneri* (15.4%, n = 16), and *L*. *weilii* (2.9%, n = 3) (Fig 1). Seven water samples (6.7%) had three or more base pair differences with any reference sequence and were not classified. Using the National Center for Biotechnology Information (NCBI) Basic Local Alignment Search Tool (BLAST), the unclassified were confirmed to be pathogenic *Leptospira*. The seven unclassified sequences matched with pathogenic *Leptospira* in the NCBI BLAST database at 97% or above as either *L*. *interrogans* (n = 6) or *L*. *borgpetersenii or L*. *kirschneri* (n = 1). The majority of rodent samples were most similar to *L*. *kirschneri* (70.6%, 12/17 rodents), followed by *L*. *borgpetersenii* (11.8%, 2/17 rodents), *L*. *interrogans* (11.8%, 2/17 rodents) and one sample was unclassified (5.9%, 1/17). The sequences from the cow, horse, and from one of the human cases were most similar to *L*. *interrogans*, while the sequence from the second human case aligned most closely with *L*. *kirschneri* (Fig 1, S1 Fig).

**Fig 2:**
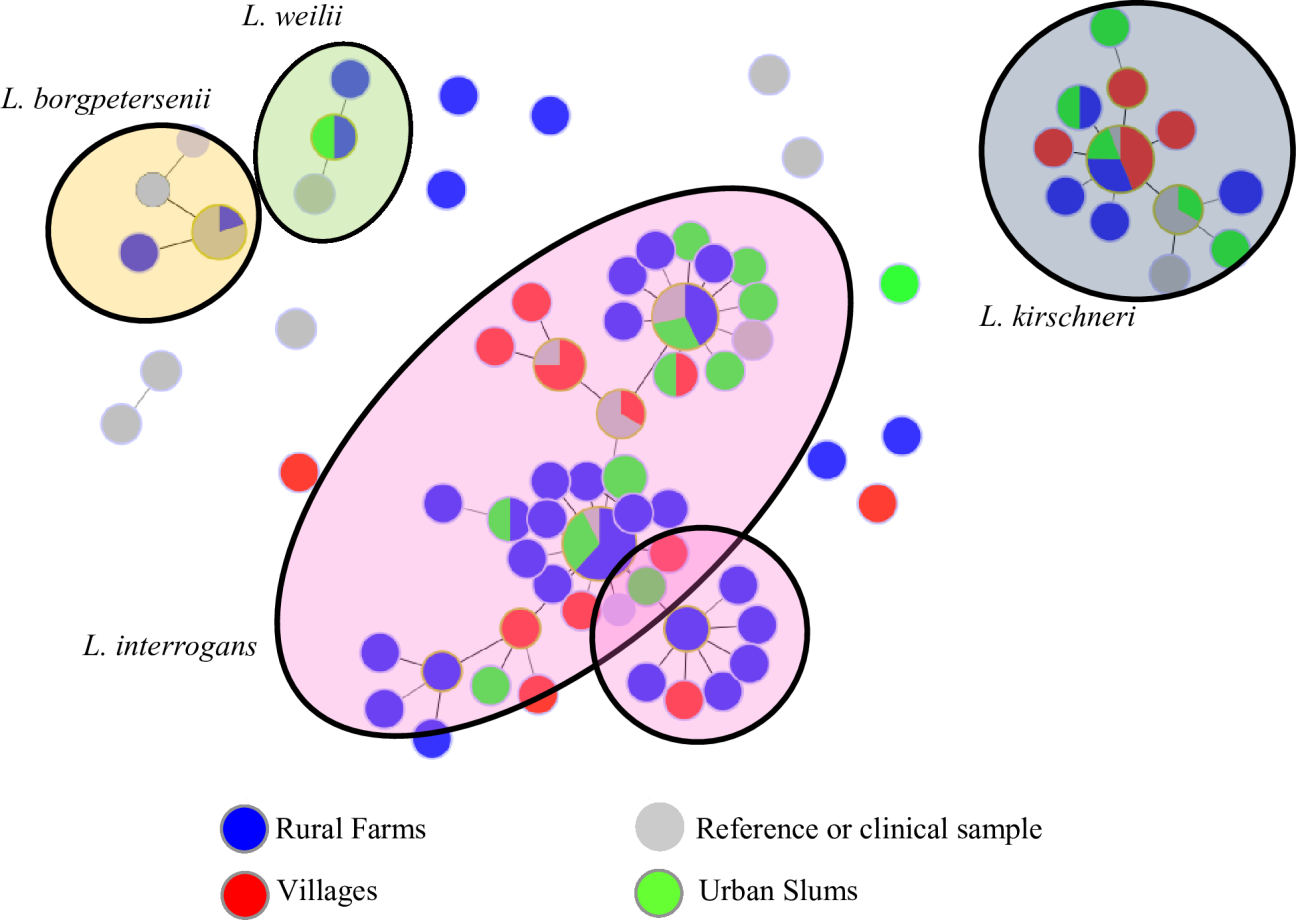
Minimum Spanning Trees of *Leptospira*-positive secY sequences from surface water samples (n = 104), rodent samples (n = 17), and clinical samples (n = 4) by community type. Community type of origin of water and rodent sequences are color coded. *L*. *interrogans*, *L*. *kirschneri* and *L*. *weilii* were found in water samples from all community types. *L*. *borgpetersenii* was found in rodents from rural farms but not in water samples.

**Fig 3:**
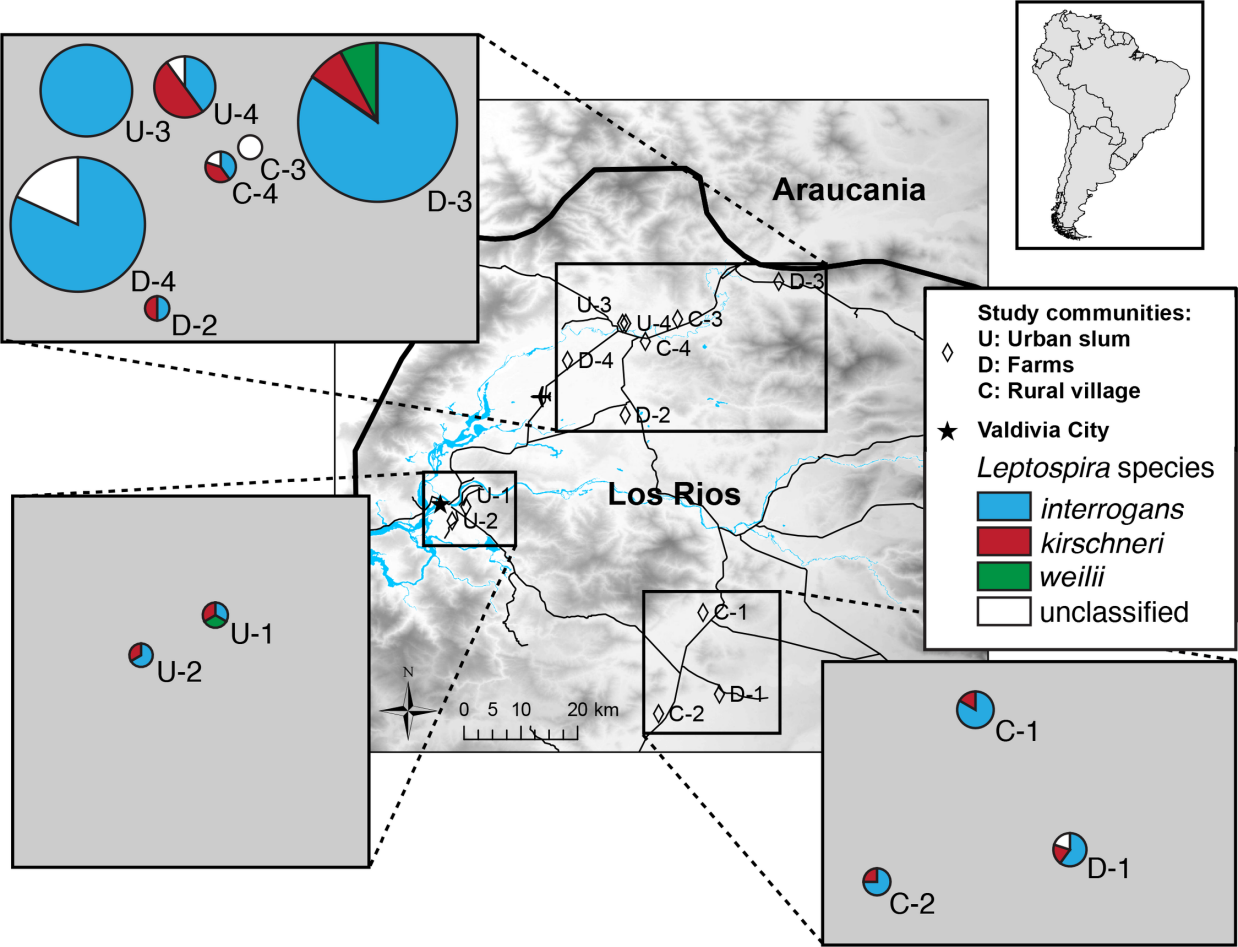
Community-level distribution of *Leptospira* species from water samples collected from households in Los Rios Region, Chile. Communities corresponded to urban slums (U), rural villages (C), and farm communities (D). Size of the pie correlates with the number of sequences available for the community.

Community type was not associated with the presence of a particular *Leptospira* species (p-value = 0.27) (Table 2). A visual representation is seen in Fig 2 where the community types are equally distributed across the three known *Leptospira* species in the MST produced in PHYLOViZ. However, at the community level, there were notable differences in distribution of species (Fig 3). Community D-4, seemed to have *L*. *interrogans* only; 19 of 22 samples were classified as *L*. *interrogans*, and the other three, while unclassified, were also suggested to be *L*. *interrogans* based on results from the NCBI BLAST. Similarly, all sequences from community U-3 (n = 15) were classified as *L*. *interrogans*. In other communities, diversity was evident despite the small number of sequences. In community U-1, for example, among the three samples sequenced, all three *Leptospira* species were identified (*L*. *interrogans*, *L*. *kirschneri* and *L*. *weilii*).

**Table 2:** *Leptospira* species phylogenetic classification of *secY* (202 bp) sequences obtained from water samples collected from the peri-domestic environment and sample/household characteristics.

	*L*. *interrogans*	*L*. *kirschneri*	*L*. *weilii*	Total	p-value
**Sample Type**					0.21
Animal drinking trough	8 (88.9%)*	1 (11.1%)	0	9	
Puddle	42 (77.8%)	11 (20.4%)	1 (1.9%)	54	
Human drinking water	5 (55.5%)	2 (22.2%)	2 (22.2%)	9	
Flowing source	8 (100%)	0	0	8	
Container	15 (88.2%)	2 (11.8%)	0	17	
**Year of Sample**					0.07
Year 1	15 (65.2%)	7 (30.4%)	1 (4.4%)	23	
Year 2	63 (85.1%)	9 (12.2%)	2 (2.7%)	74	
**Season of Sample**					0.02
Spring (August-November)	43 (86.0%)	4 (8.0%)	3 (6.0%)	50	
Summer (December-April)	35 (74.5%)	12 (25.5%)	0	47	
**Community Type**					0.25
Farms	46 (86.8%)	5 (9.4%)	2 (3.8%)	53	
Rural villages	10 (71.4%)	4 (28.6%)	0	14	
Urban slums	22 (73.3%)	7 (23.3%)	1 (3.3%)	30	
**Average Temperature Past 7 days**					0.07
<7°C	17 (94.4%)	0	1(5.6%)	18	
7–14°C	30 (76.9%)	7 (17.9%)	2 (5.1%)	39	
>14°C	31 (77.5%)	9 (22.5%)	0	40	
**Average Rainfall Past 30 days**					0.12
<50mm	38 (76.0%)	11 (22.0%)	1 (2.0%)	50	
50-100mm	22(78.6%)	5 (17.9%)	1 (3.6%)	28	
>100mm	18 (94.7%)	0	1 (5.3%)	19	
**Presence of Seropositive Dogs ǂ**					1.0
No	11 (78.6%)	3 (21.4%)	0	14	
Yes	27 (77.1%)	7 (20.0%)	1 (2.9%)	35	
**Presence of Seropositive Livestock ǂ**					0.04
No	12 (66.7%)	5 (27.8%)	1 (5.6%)	18	
Yes	21 (95.5%)	1 (4.5%)	0	22	
**Presence of PCR Positive Rodents ǂ**					1.0
No	8 (88.9%)	1 (11.1%)	0	9	
Yes	70 (79.5%)	15 (17.0%)	3 (3.4%)	88	

* Percentages correspond to the distribution of *Leptospira* species for each level of the variable investigated.

**ǂ** Results correspond to sequences from households where both sequences and dogs, livestock, and rodents, respectively, were present.

In five communities (two rural villages and three farm areas) where *L*. *kirschneri* was found in rodents, it was also found in at least one water sample from the respective community. *L*. *kirschneri* was found in a rodent from an additional rural village but only one *secY* water sequence (classified as *L*. *interrogans*) was available for comparison. *L*. *interrogans* was found in two rodents (one from a rural community and one from a rural village) but it is also the most commonly found species in water samples across all communities. Sequences classified as *L*. *borgpetersenii* were found in two rodents but in none of the water samples (Fig 1, S1 Fig). The diversity of *Leptospira* species in water samples in a community did not appear to be associated with the proportion of infected animals in the community (S2 Fig), and in general, *L*. *interrogans*, *L*. *kirschneri*, and *L*. *weilii* were found across the various communities (Fig 3).

### Sample characteristics associated with Leptospira diversity

Samples that were considered “not classified” in the MST procedure were excluded from this analysis. Sequences obtained in summer corresponded to a higher proportion of *L*. *kirschneri* (25.5%, 12/47) compared to sequences obtained in the spring (8.0%, 4/50), and all three *L*. *weilii* samples were obtained in the spring (Table 2, p-value = 0.02). In the subset of households with livestock, the water sequences from households with seropositive livestock contained a higher proportion of *L*. *interrogans* (95.4%, 21/22) than water samples from households with seronegative livestock (66.7%, 12/18 of *L*. *interrogans*) (p-value = 0.04).

The type of water sample did not appear to be associated with the presence of a particular *Leptospira* species (p-value = 0.24), but all the sequences from flowing water sources (n = 8) were most closely related to *L*. *interrogans* (Table 2). The temperature in the preceding week, and rainfall in the preceding month of sample collection were also not statistically significantly associated with the distribution of presumptive *Leptospira* species (p-value = 0.07 and p-value = 0.12, respectively). Nonetheless, 94.4% (17) of samples were identified as *L*. *interrogans* when the average temperature was below 7°C, while 76.9% (30) and 77.5% (31) of samples were identified as *L*. *interrogans* at temperatures between 7°C and 14°C or above 14°C, respectively (Table 2). *L*. *interrogans* was also more common at higher levels of precipitation with 94.7% (18) of samples identified when the rainfall in the preceding month was above 100 mm, compared to 78.6% (22 samples) when rainfall was between 50 and 100mm, and 76.0% (38 samples) when rainfall was less than 50 mm in the preceding month (Table 2).

## Discussion

This study reports the presence of pathogenic *Leptospira* in the peri-domestic environment of households in several community types, and the substantial differences in *Leptospira* diversity at the community level. The development of PCR methods for detecting pathogenic *Leptospira* over the past decade has greatly improved the efficiency with which a specific site can be tested for *Leptospira* presence. Several studies have noted the success of these methods in establishing the presence of the bacteria in bodies of water such as rivers, canals and streams [21,36,37]. Fewer studies have examined the presence of pathogenic *Leptospira* in the daily human environment where regular contact with contaminated water sources may occur. In Peru, researchers examined surface water samples from puddles and gutters in an urban location, and wells, fish farms and streams in rural areas. *Leptospira* was detected in both urban and rural areas, but with higher concentration and more frequent contamination in the urban compared to the rural sites [19]. More frequent contamination with pathogenic *Leptospira* was also observed in urban sites compared to rural areas in India [38]. In our study, greater differences in the proportion of water samples testing positive were observed at the community level than across community types (urban slums, rural villages or farms). This is indicative of the importance of small scale transmission dynamics in the ecology of leptospirosis.

This study also describes the difference in pathogenic *Leptospira* species obtained in environmental water samples in different communities, and across community types. Results in Peru suggested that non-*L*. *interrogans* species were more likely in samples from rural areas than from urban sites due to the presence of more animal species [19]. It is reasonable to expect a higher presence of non-*L*. *interrogans* species in locations where there is a higher diversity and density of animal species overlapping in the same environment; however, this was not generally reflected in our findings where the majority of sequences in rural areas were classified as *L*. *interrogans* (86.8%), which was higher than in urban sites (73.3%) (Table 2). This was primarily driven by the large number of samples with *L*. *interrogans* in farm communities D-3 (84.6%) and D-4 (86.4%). Furthermore, in some urban sites, U-1 for example, all three *Leptospira* species were present (*L*. *interrogans*, *L*. *kirschneri*, and *L*. *weilii*). A possible explanation of these findings is that the urban slum communities in the study were in close proximity to rural farms, and wild mammals may transmit these more diverse *Leptospira* species between the rural and urban areas. Nevertheless, the variation in diversity across all twelve communities highlights the importance of better understanding local transmission dynamics for assessing *Leptospira* risk in the peri-domestic environment. The seasonality of leptospirosis has been well-described; human and animal incidence increases in periods following warm temperatures and heavy rainfall [13,39]. Temporal patterns in species diversity was reported in a study in New Caledonia which found that black rats normally carried *L*. *interrogans*, but during the hot season, they were found to carry *L*. *borgpetersenii* [40]. In this study, most of the sequences corresponded to *L*. *interrogans* but, among water samples collected in spring, there was increase in the proportion that were classified as *L*. *kirschneri* compared to sequences obtained in the summer (Table 2). As a cross-sectional study with sampling that took place over two years, we cannot separate the effect of community differences from a potential true season effect. Future prospective studies can examine the joint influence of rainfall, temperature, and host population dynamics on the *Leptospira* diversity and determine seasonality patterns.

*L*. *interrogans* was more common in households with seropositive livestock (sheep or cattle) than in households without seropositive livestock (Table 2), which is consistent with reports of sheep and cattle carrying *L*. *interrogans* in Chile [41] and in other animal species and rural water sources in other geographic locations [42–44]. Considering the large number of sequences classified as *L*. *interrogans*, it also possible that sequencing of the *secY* target may not provide enough discrimination to capture associations between other examined factors and molecular diversity. Alternatively, it is possible that water samples were contaminated with more than one *Leptospira* species, and that *L*. *interrogans* was more commonly amplified, since PCR is a competitive process that amplifies the most abundant species in the sample. This would result in less diversity than there really was mainly because the relative concentration of non-*L*. *interrogans* species is low. Furthermore, presence of multiples species in a sample could be a factor leading to the many sequences that were not able to be classified. The impact of contamination by multiple species on PCR detection and subsequent genotyping needs further investigation Several methods have been developed for genetic classification of *Leptospira* isolates[45–48]; however, being able to use culture-free methods is essential for large-scale and systematic monitoring of environmental contamination. Recently, a culture-free high resolution melting method was proposed for identification of *Leptospira* strains in blood samples at the species and subspecies levels [47]. Similar methods need to be developed and optimized for environmental testing.

A limitation of culture-free methods for identification of *Leptospira* in the environment is the inability to differentiate between live and dead bacteria [49]. While the detection of DNA in the environment may not directly represent infection risk, the benefit of being able to test a large number of samples and obtain fast results may outweigh this limitation for the purpose of obtaining an indicator of contamination level and of the genetic makeup of the circulating strains. With the development of recent methods that target RNA and DNA in environmental samples, it may be possible to discern not only presence of *Leptospira* but also the relative abundance of viable *Leptospira* [45,46,50]. We expect that both detection limit, a likely problem when testing environment sample, and testing of mixed DNA contributed to our inability to sequence all the PCR positive samples or classify some of the s*ecY* sequences. Upon further review, seven of the eight unclassified samples (seven water samples, and one rodent sample) had some ambiguous base pairs in their chromatograms and the eighth sample had gaps at the end of the sequence where the final bases of the primer should have been. All eight sequences matched with *L*. *interrogans* in the NCBI BLAST database with at least 97% similarity. There were an additional 49 samples that were not included in this analysis because of the poor quality of the chromatograms. It is important to note that all these samples were detected as pathogenic *Leptospira* by at least one of the two detection protocols.

**Fig 4:**
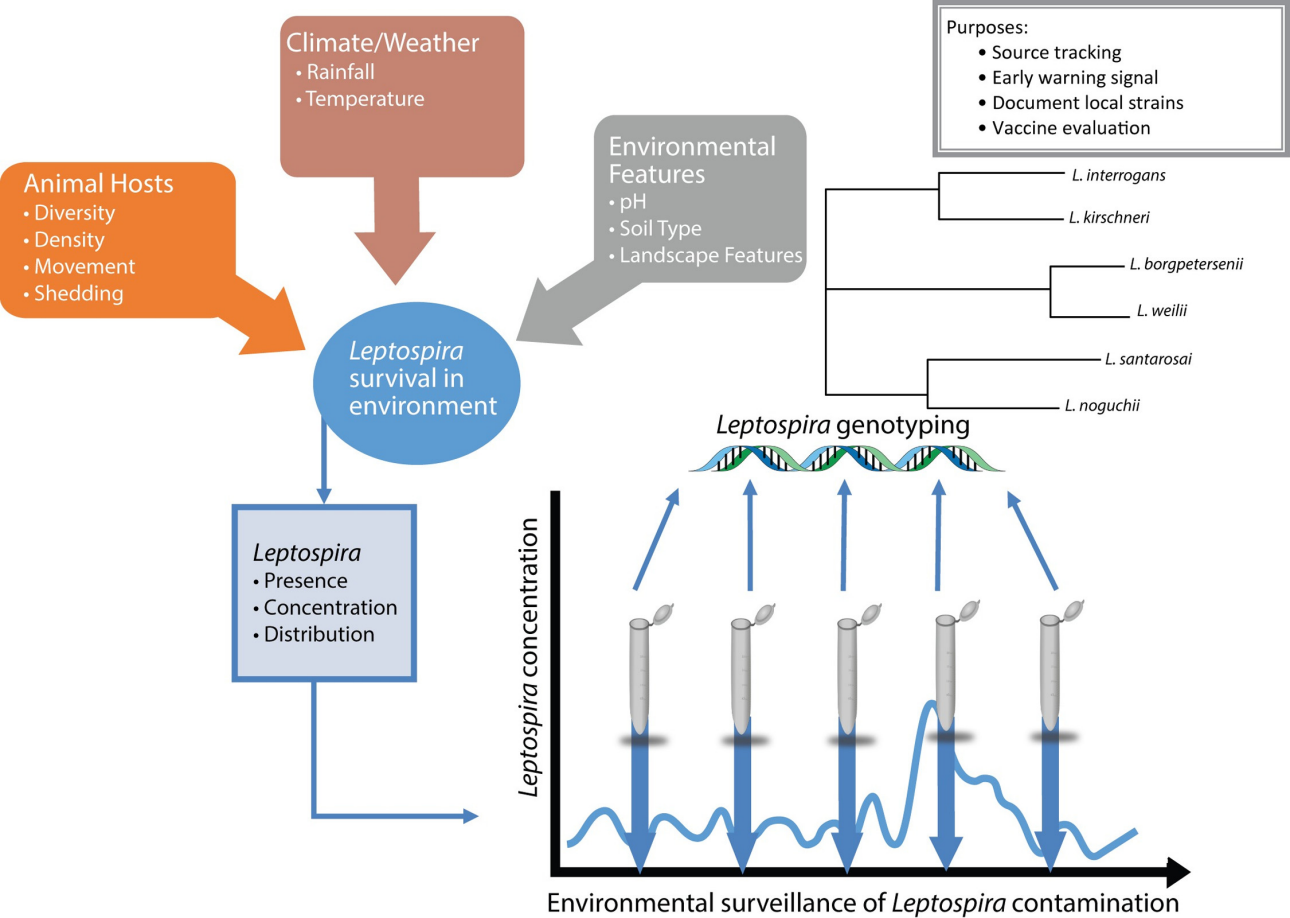
Diagram of the theoretical framework for environmental surveillance of *Leptospira*. Systematic sampling to obtain changes in presence, concentration and genotype of *Leptospira* could aid in detecting changes in the local epidemiology, including shifts in genetic diversity and potential increases in contamination that may lead to outbreaks.

The environment is recognized as an important source of infection and development of proper methods for surveillance of *Leptospira* in soil and water can provide useful information for research and public health programs. An ideal environmental surveillance program may include detection and quantification of pathogenic *Leptospira* in environmental samples in a geographical area, followed by molecular classification and comparison with local strains from human and animal cases (Fig 4). Quantification of *Leptospira* contamination using qPCR methods, was beyond the original scope of this study, but would allow more accurate monitoring of high risk areas and environmental risk assessment [19]. This ideal environmental surveillance program could integrate human and animal genotyping data with the results of environmental sampling to allow for tracking the source of infections. Additionally, changes in the diversity of *Leptospira* detected may indicate a shift in the local epidemiology in the various animal hosts or lapses in control programs [44]. Monitoring changes in the level of contamination at appropriate scales, along with environmental features such as rainfall, temperature, pH, soil type, and geographic features [38,47] could potentially aid in outbreak prediction. This concept of environmental surveillance has been used to assess the safety of recreational waters for swimming in the United States. The primary pathogen observed is *E*. *coli*, where a protocol was developed to predict dangerously high concentrations in the environment. The model includes measurements pertaining to environmental conditions (wave height and log_10_turbidity), weather conditions (past 48 hour rainfall and wind direction), and animal factors (the presence of birds). After four years’ worth of *E*. *coli* measurements taken from the water source at least four consecutive days per week, the model was able to predict unsafe swimming days with 90.2–97.5% specificity, and 36.4–59.1% sensitivity [51]. Further development of laboratory methods is still needed to allow for a similar systematic, large-scale, and cost-effective identification of *Leptospira* in the environment.

## Conclusion

This study demonstrated that PCR methods can be used to assess the presence and species diversity of pathogenic *Leptospira* in surface waters in several community settings. Presence and diversity of *Leptospira* species varied substantially at the community level, more than by community type, suggesting that targeted prospective monitoring may be appropriate to identify local mechanisms responsible for enhanced transmission, including critical periods of high risk, and key animal hosts.

## Supporting Information

S1 Fig Neighbor-joining tree of 104 water samples, 17 rodent samples, 20 reference strains and four clinical samples, from urban, rural village, and farm communities in the Los Rios Region, Chile, 2010–2012 using the secY gene. Samples labeled with a *Leptospira* species name are reference strains. Samples are coded to represent their community type of origin (C: rural village, D: farms, U: urban slum) and whether it is a water (B) or rodent (R) sequence. The clinical samples are listed by their types: Clinical-Human (blood sample), Human-Urine, Horse-Urine, and Cow-Blood.
(PDF)

S2 Fig Community-level prevalence of *Leptospira* in dogs, livestock and rodents and distribution of *Leptospira* species obtained from water samples in the same communities. Dogs and livestock show estimates of seroprevalence by MAT and rodents show the PCR positive proportion. Community types are represented by C: rural village, D: farms, and U: urban slum.
(PDF)

S1 Table List of reference *Leptospira* species used in the phylogenetic analysis. (DOCX)
