# Supplementary figures and images for: Distribution and Diversity of Pathogenic Leptospira Species in Peri-domestic Surface Waters from South Central Chile
Source: PLoS Negl Trop Dis. 2016 Aug 16;10(8):e0004895. doi: 10.1371/journal.pntd.0004895 (PMC4986978; doi:10.1371/journal.pntd.0004895)

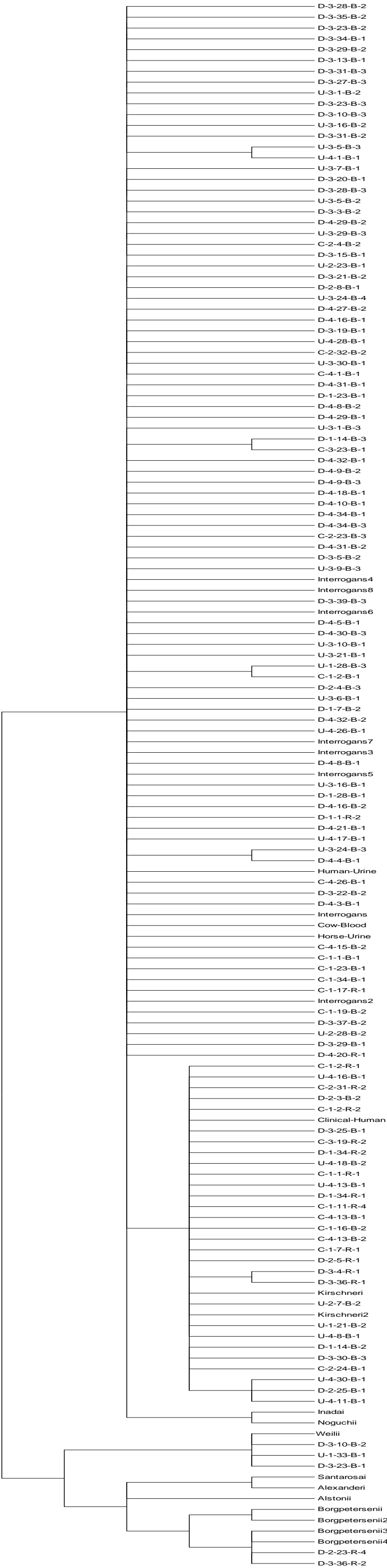

Supplement: S1 Fig — Samples labeled with a Leptospira species name are reference strains. Samples are coded to represent their community type of origin (C: rural village, D: farms, U: urban slum) and whether it is a water (B) or rodent (R) sequence. The clinical samples are listed by their types: Clinical-Human (blood sample), Human-Urine, Horse-Urine, and Cow-Blood. (PDF) [file pntd.0004895.s001.pdf]
