# Supplementary material for: Distribution and Diversity of Pathogenic Leptospira Species in Peri-domestic Surface Waters from South Central Chile
Source: PLoS Negl Trop Dis. 2016 Aug 16;10(8):e0004895. doi: 10.1371/journal.pntd.0004895 (PMC4986978; doi:10.1371/journal.pntd.0004895)
Supplement: S2 Fig — Dogs and livestock show estimates of seroprevalence by MAT and rodents show the PCR positive proportion. Community types are represented by C: rural village, D: farms, and U: urban slum. (PDF) [file pntd.0004895.s002.pdf]

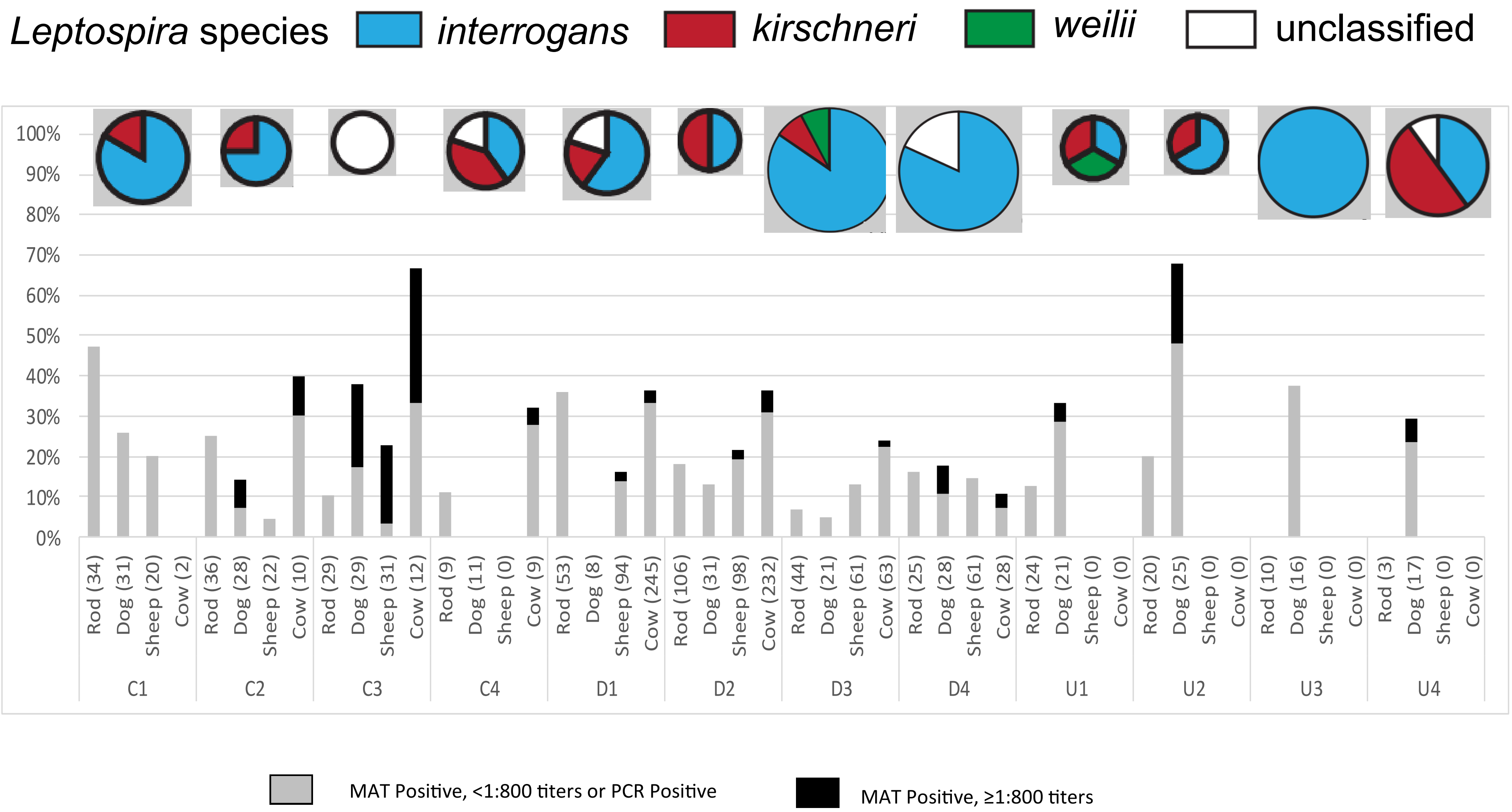

Rodents were trapped and tested by PCR as described in Munoz-Zanzi et al. (2014). Dogs, sheep and cows owned by the households were tested for *Leptospira* antibodies using a microscopic agglutination test (MAT) carried out at the Centers for Disease Control, United States as described in Lelu et al. (2015). A panel of 20 serovars from 17 serogroups was used including serovars Australis, Bratislava, Autumnalis, Bataviae, Canicola, Djasiman, Grippotyphosa, Icterohaemorrhagiae, Mankarso, Pomona, Pyrogenes, Wolffi, Ballum, Javanica, Tarassovi, Cynopteri, Borincana, Alexi, Georgia, and Celledoni. For each serovar, samples were identified as positive with titers of 1:100 or higher. Final reported titers were the highest dilution of serum that agglutinated at least 50% of the cells.
