# Supplementary material for: Distribution and Diversity of Pathogenic Leptospira Species in Peri-domestic Surface Waters from South Central Chile
Source: PLoS Negl Trop Dis. 2016 Aug 16;10(8):e0004895. doi: 10.1371/journal.pntd.0004895 (PMC4986978; doi:10.1371/journal.pntd.0004895)
Supplement: S1 Table — (DOCX) [file pntd.0004895.s003.docx]

S1 Table. Selection of *Leptospira* reference strains, representing a variety of species, used for phylogenetic analysis. Isolates were obtained from the WHO Reference Center, Royal Tropical Institute.

|  | Species | Serovar |
| --- | --- | --- |
| 1 | *L.interrogans* | Hardjo type Prajitno |
| 2 | *L.alexanderi* | Yunnan |
| 3 | *L.inadai* | Mangus |
| 4 | *L.kirschneri* | Bim |
| 5 | *L.borgpetersenii* | Hardjo type Bovis |
| 6 | *L.weilii* | Celledoni |
| 7 | *L.noguchii* | Argentiniensis |
| 8 | *L.santarosai* | Goiano |
| 9 | *L.alstonii (genomospecies1)* | Pinchang |
| 10 | *L.kmetyi* | Malaysia |
| 11 | *L.interrogans* | Bratislava |
| 12 | *L.interrogans* | Autumnalis |
| 13 | *L.borgpetersenii* | Ballum |
| 14 | *L.interrogans* | Canicola |
| 15 | *L.kirschneri* | Grippotyphosa type Moskva |
| 16 | *L.interrogans* | Icterohaemorrhagiae |
| 17 | *L.borgpetersenii* | Javanica |
| 18 | *L.borgpetersenii* | Tarassovi |
| 19 | *L.interrogans* | Wolfii |
| 20 | *L.interrogans* | Pyrogenes |
| 21 | *L.interrogans* | Pomona |
